# Refractory Chylothorax in a Patient With Severe Mitral Regurgitation

**DOI:** 10.1002/rcr2.70229

**Published:** 2025-06-16

**Authors:** Sheena Jing Yi Ng, Qiao Li Tan, Ken Junyang Goh, Si Ling Young, Brian Lee Wei Chua, Jane Jing Yi Wong, Ivana Gilcrist Phua, Wen Ting Lim, Carrie Kah‐Lai Leong

**Affiliations:** ^1^ Department of Respiratory & Critical Care Medicine Singapore General Hospital Singapore Singapore

**Keywords:** chylothorax, mitral regurgitation, pleural effusion

## Abstract

We report a case of chylothorax secondary to severe mitral regurgitation, in the absence of heart failure with a low ejection fraction. A 49‐year‐old male presented with mitral valve infective endocarditis, complicated by severe mitral regurgitation with persistent vegetations 5 weeks into treatment. He declined valvular repair. He was admitted four times in the following year for symptomatic right pleural effusion requiring therapeutic chest drainage. Pleural fluid was milky, with elevated triglyceride levels consistent with chylothorax. He underwent mitral valve replacement, with no recurrence of chylothorax to date. The mechanism of chylothorax in mitral regurgitation is likely due to high central venous pressure, with transmitted high right lymphatic duct and thoracic duct pressures. This may then cause reflux of lymph and chyle into pleural lymphatics and subsequent leakage of fluid into the pleural space. This case illustrates mitral regurgitation as a rare cause of chylothorax, with mitral valve replacement granting remission.

## Graphical abstract

We report an unusual case of refractory chylothorax secondary to severe mitral regurgitation, with mitral valve replacement granting remission. We explore potential pathophysiological mechanisms and therapeutic options. This case highlights that treatment of the underlying cause is key to the successful management of chylothoraces.

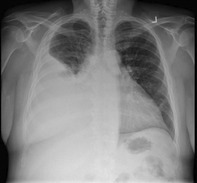

## 1 Introduction

Chylothorax occurs as a result of disruption to the flow of chyle through the thoracic duct, which may result from obstruction due to compression, trauma, anatomical anomalies and malformations, or translocation of chylous fluid from the abdominal compartment. The majority of cases of chylothorax are due to malignancy and trauma [1]. We report a case of chylothorax secondary to severe mitral regurgitation, in the absence of heart failure with a low ejection fraction. This is the first reported case to the best of our knowledge.

## 2 Case Report

A 49‐year‐old man presented with native mitral valve infective endocarditis, with disseminated *Streptococcus anginosis* bacteremia. He has a past medical history of hypertension, hyperlipidemia and diabetes mellitus. This was complicated by severe mitral regurgitation and septic emboli to the brain, lungs, spleen and kidneys. Transthoracic echocardiogram showed a 19 by 13 mm vegetation on the anterior mitral valve, with severe mitral regurgitation. Left ventricular ejection fraction was preserved at 60%–65%. He developed acute pulmonary edema requiring endotracheal intubation and mechanical ventilation in the intensive care unit and was extubated after 2 days. In spite of effective antibiotics, persistent vegetations were present on transthoracic echocardiogram 5 weeks into treatment. He was offered surgery to remove infected tissue and for valvular repair but declined. He was subsequently discharged from hospital and completed 6 weeks of antibiotics for infective endocarditis.

**FIGURE 1:**
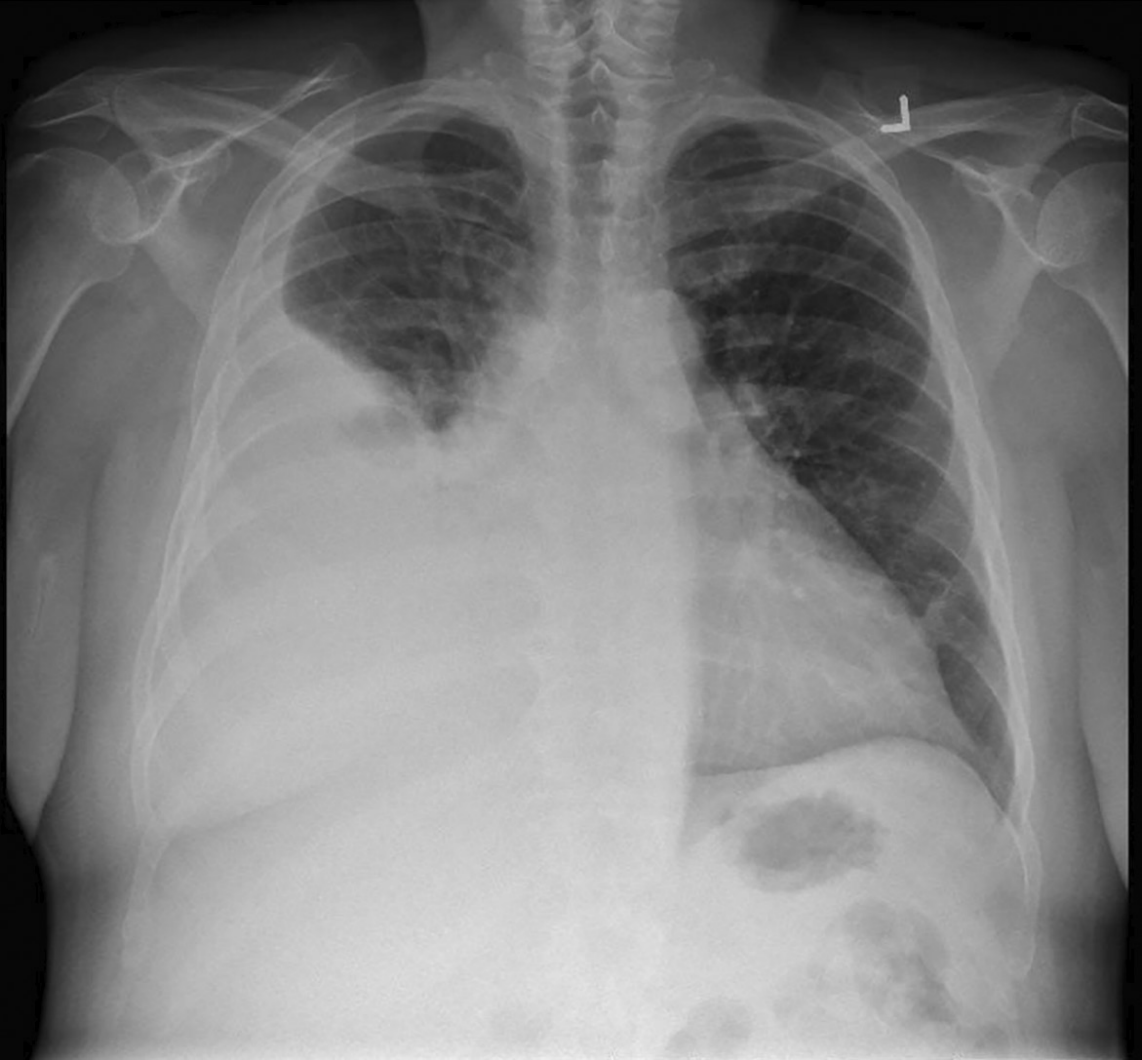
Initial chest radiograph demonstrating large right pleural effusion.

**FIGURE 2:**
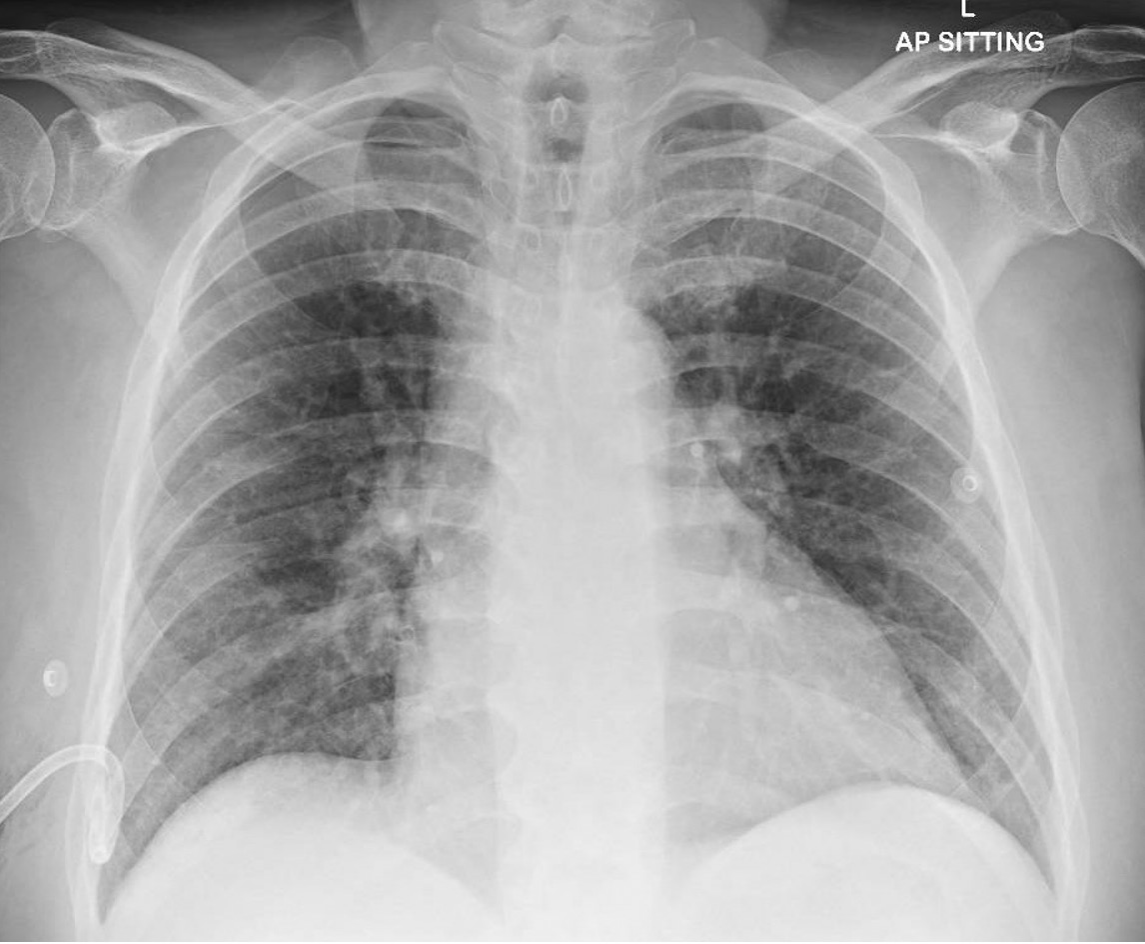
Subsequent chest radiograph demonstrating satisfactory effusion drainage following chest drain insertion.

**FIGURE 3:**
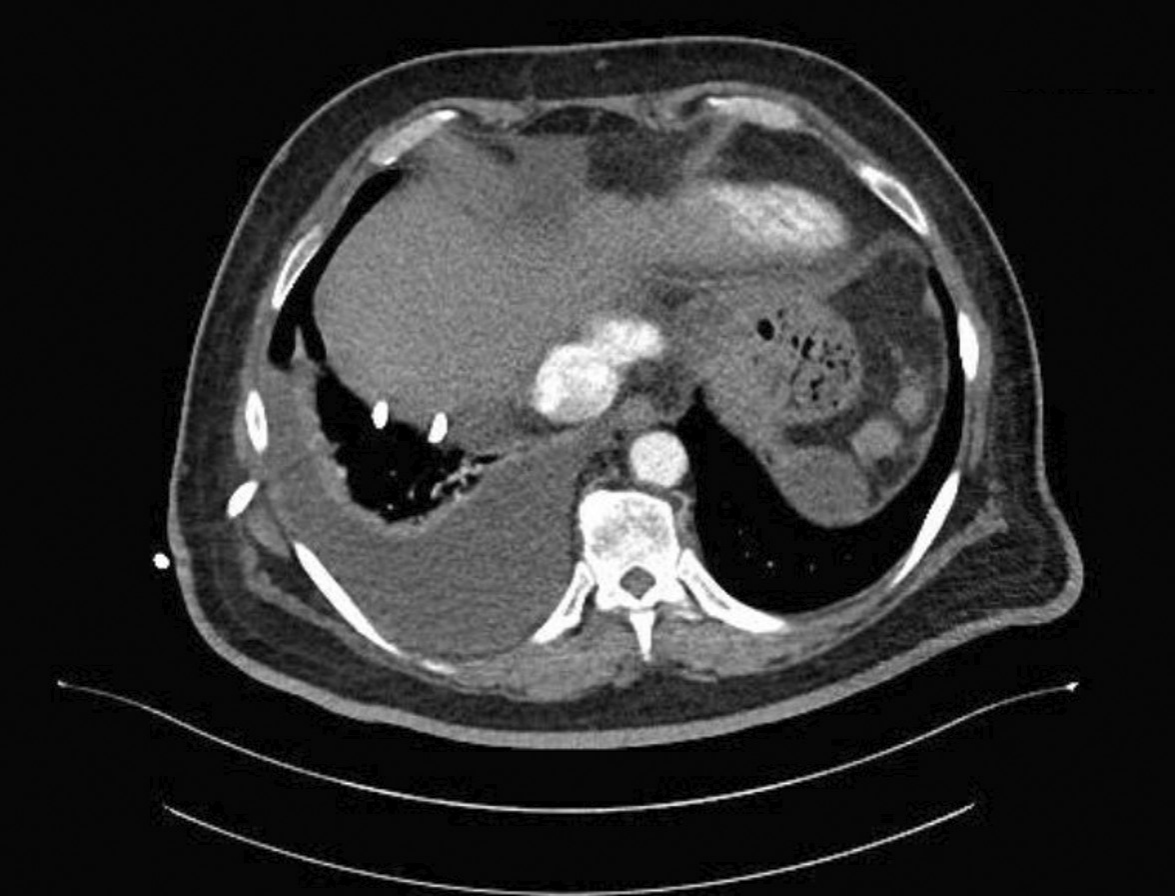
Computed tomography (CT) of the thorax demonstrating right pleural effusion with chest drain in situ. No lung mass or pleural nodularities were noted.

He was subsequently admitted four times in the following year for symptomatic right pleural effusion requiring therapeutic chest drainage (Figures 1 and 2). An average of 2–2.5 L of pleural fluid was drained at each sitting. Symptomatic recurrence of pleural effusion occurred within approximately 2 weeks to a month following chest drainage. Pleural fluid investigations were significant for a milky, lymphocytic transudate, with a triglyceride level of 1.55 mmol/L consistent with chylothorax. Pleural fluid microbiology and cytology were negative for organisms and malignant cells respectively. There was no preceding history of trauma, nor evidence of malignancy. An interval transesophageal echocardiogram was performed, which showed persistent large mobile vegetation in the mitral valve, now complicated by a large perforation, through which severe mitral regurgitation courses (Video 1). Echocardiogram also showed a resultant dilated left atrium, right ventricle and right atrium, with mild to moderate tricuspid regurgitation. Computed Tomography of the thorax showed right pleural effusion, with no suspicious lung mass, pleural nodularities or lymphadenopathy (Figure 3). An indwelling pleural catheter was discussed as an alternative option given the frequent recurrence of symptomatic pleural effusion and reluctance for surgical repair, but the patient declined.

**VIDEO 1:** Transesophageal echocardiogram demonstrating perforated mitral valve with severe mitral regurgitation. Video content can be viewed at https://onlinelibrary.wiley.com/doi/10.1002/rcr2.70229

The patient eventually agreed to surgical repair after the fourth admission for symptomatic pleural effusion requiring serial chest drainage. He underwent mitral valve replacement, tricuspid valve annuloplasty and closure of the left atrial appendage. Central venous pressure measured immediately prior to surgery was elevated at 15 mmHg. Intraoperative findings showed dilated cardiac chambers, a 1.5 cm perforation of the A2 segment of the mitral valve, and healed vegetations at P3 and the posteromedial commissure, which were excised along with the anterior mitral leaflet. Additionally, the tricuspid valve annulus was severely dilated, and tricuspid valve annuloplasty was performed. Pleural adhesiolysis was also performed.

One month following the operation, the patient presented again for symptomatic right pleural effusion requiring drainage. However, pleural fluid analysis was transudative, with a low pleural fluid triglyceride levels of 0.28 mmol/L, which was not suggestive of chylothorax. He has had no recurrence of chylothorax to date.

## 3 Discussion

This is the first reported case of chylothorax secondary to severe mitral regurgitation, in the absence of heart failure with low ejection fraction, to the best of our knowledge. Cakmak et al. reported a case of chylothorax and chylous ascites in the setting of a patient with cardiomyopathy, with a low ejection fraction of 25% and moderate mitral and tricuspid regurgitation [2]. The patient required thoracocentesis, and dietary fat restriction and optimal heart failure management showed minimal response [2]. Chylothorax has also been reported in the setting of severe rheumatic mitral stenosis [3]. In this case report, there was one episode of recurrence of chylothorax 2 weeks following mitral valve replacement, but no subsequent recurrence since [3].

The mechanism of chylothorax in mitral regurgitation is likely due to high central venous pressure, with transmitted high right lymphatic duct and thoracic duct pressures. The resultant increased hydrostatic pressure may then cause reflux of lymph and chyle into the pleural lymphatics and subsequent leakage of fluid into the pleural space. It is unclear why our patient presented with only right pleural effusion, without left pleural effusion or chylous ascites. During the first admission for infective endocarditis, the patient underwent uncomplicated insertion of a right peripherally inserted central catheter (PICC). Although PICC insertion may have caused inadvertent injury to the right bronchomediastinal trunk or right lymphatic duct during catheter insertion and contributed to a degree of central vein stenosis, there was no evidence of this seen on CT imaging.

Identification and treatment of the underlying cause is key to successful management of chylothoraces. General management principles include diagnostic and therapeutic pleural fluid drainage and a low‐fat diet with medium‐chain triglycerides. Other options for refractory chylothorax include pleurodesis, somatostatin, and octreotide, thoracic duct embolization, surgical thoracic duct ligation or repair [4]. Prolonged drainage may be harmful due to continued loss of proteins, immunoglobulins, T‐lymphocytes into the pleural space, leading to relative immunocompromise. Other complications include malnutrition and electrolyte disturbances, hemodynamic instability, overall contributing to increased morbidity and mortality [5]. Our case illustrates identification of mitral regurgitation as a rare cause of refractory chylothorax, with eventual mitral valve replacement granting remission.

## Author Contributions

All authors were involved in the review and editing of the manuscript. Dr. Ng and Dr. Leong were involved in the writing of the original manuscript. Dr. Ng, Dr. Goh and Dr. Leong were involved in the administration of care to the patient.

## Ethics Statement

The authors declare that written informed consent was obtained for the publication of this manuscript and accompanying images using the consent form provided by the Journal.

## Conflicts of Interest

The authors declare no conflicts of interest.

## Data Availability

Data sharing is not applicable to this article as no new data were created or analyzed in this study.
